# Therapeutic hypothermia for neonates: a bibliometric analysis and visualization research

**DOI:** 10.3389/fneur.2025.1565749

**Published:** 2025-05-07

**Authors:** Tingting Zhou, Peishan Liang, Na Dai, Mingxin Ma, Jiangang Lin, Jie Zhu, Guangli Ren

**Affiliations:** ^1^Department of Pediatric, General Hospital of Southern Theater Command of PLA, Guangzhou, China; ^2^Graduate School, Guangzhou University of Chinese Medicine, Guangzhou, China; ^3^The First School of Clinical Medicine, Southern Medical University, Guangzhou, China

**Keywords:** therapeutic hypothermia, neonates, bibliometric, CiteSpace, VOSviewer

## Abstract

**Background:**

Therapeutic hypothermia is an important treatment for cerebral protection and has a positive effect on neonatal encephalopathy. This study aims to analyze the research hotspots and frontiers of therapeutic hypothermia for neonates through bibliometric analysis and visualization research.

**Methods:**

The articles and reviews on therapeutic hypothermia for neonates were identified from the Web of Science Core Collection (WOSCC) database on October 18, 2024. CiteSpace and VOSviewer were used to analyze the countries/regions, institutions, authors, journals, references, and author keywords.

**Results:**

A total of 1,199 articles were retrieved from 378 institutions in 75 countries/regions. The annual number of publications and citations showed an upward trend in this field. Massaro, An N. N., and Shankaran. S. were the key authors who had most publications and citations. *Pediatric Research* was the most popular journal in the field, *Pediatrics* was the most influential. All the author keywords were divided into 12 clusters, and “hypoxia-ischemia encephalopathy,” “therapeutic hypothermia” and “perinatal asphyxia” were high-frequency keywords in this field. Keyword burst shows that “childhood outcome,” “neonatal seizure,” “preterm,” and “risk factors” were important aspects of research in recent years.

**Conclusion:**

The present study is the first to apply bibliometric analysis to explore therapeutic hypothermia for neonates, aiming to identify research hotspots and frontiers in this field. In recent years, research on therapeutic hypothermia for neonates has rapidly increased, with therapeutic hypothermia for perinatal asphyxia neonates being a research hotspot. To improve the neurological prognosis of neonates, more researches focused on how to expand the benefits of the neonatal population and enhance neuroprotective effects. It may provide future research directions for neonatal experts.

## 1 Introduction

Therapeutic hypothermia (TH) has been used as a neuroprotective treatment for patients with brain injury since the 1980s (1). It plays a neuroprotective role by maintaining the core temperature at 33 ~ 34°C, slowing down brain metabolism, reducing oxygen use, and reducing oxygen-free radical production (2, 3). However, neonates also face the problem of neurological damage, which is mainly caused by perinatal asphyxia (4). The global incidence of neonatal hypoxic–ischemic brain damage has been reported to range from 1 to 8 per 1,000 live births (5, 6). It is a major cause of neonatal death and permanent consequences (7). Pediatric specialists began to research the effect of TH for neonates in the hope of reducing mortality and improving neurodevelopmental outcomes.

The study of bibliometrics integrates mathematics, statistics, and philology, and examines all knowledge carriers quantitatively (8). It can not only make quantitative analysis and qualitative analysis of countries/regions, institutions, journals and authors, but it also can evaluate and predict research hotspots and frontiers (9). Compared with reviews and meta-analyses, bibliometrics can better reflect the research status and development trends of the research. It is suitable for analyzing the literature of TH for neonates, currently, there are no studies related to the bibliometric analysis of this topic.

In the present study, we used CiteSpace and VOSviewer, two commonly used bibliometric tools, to analyze the research status of TH for neonates and to draw the knowledge map. It can provide reference information in this field for neonatal researchers.

## 2 Materials and methods

### 2.1 Data collection

**Figure 1:**
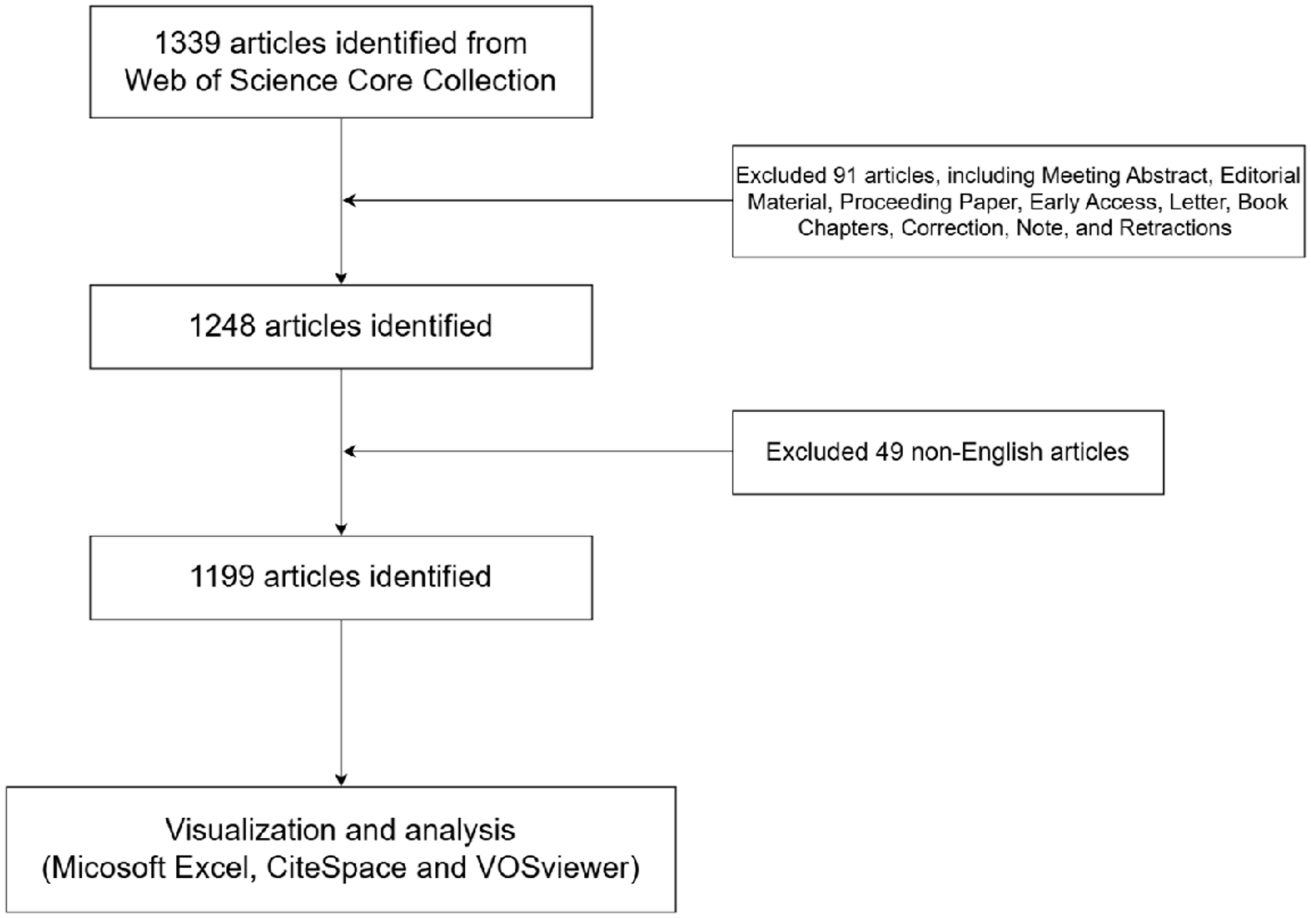
Flow chart of literature retrieval, screening and analysis.

Web of Science is the leading research platform for accessing information in hard sciences, social sciences, arts, and humanities, and it is also the independent global citation database of the world’s most reliable publishers (10). To increase the data’s representativeness and accessibility, an online literature search was conducted in the Web of Science Core Collection (WOSCC) databases, which is widely used in bibliometric analysis due to its comprehensive citation indexing and high-quality data. Thesauruses of TH were determined through searching the Medical Subject Headings (MeSH) database^1^ and added to the search query, as follow: [TI = (therapeutic hypothermia) OR AB = (therapeutic hypothermia) or AK = (therapeutic hypothermia)] AND [TI = (newborn or neonate) OR AB = (newborn or neonate) or AK = (newborn or neonate)]. In order to achieve more accurate results, the search targeted the “Title,” “Author keyword,” and “Abstract.” The articles have been obtained after selecting article or review article based on their literature type and excluding non-English literature. The included articles were cross-checked by the two independent researchers who examined the title, abstract, keywords etc. If there was disagreement, a third researcher was invited to participate in the discussion and read the full text to decide. The literature search was completed on October 18, 2024. A total of 1,339 articles were retrieved; 91 articles were excluded by limiting the article type, and 49 articles were excluded by limiting the language. In the end, 1,199 articles were exported as full records with cited references in the format “download_txt.” (Figure 1).

### 2.2 Data analysis

CiteSpace is a Java-based information visualization software developed by Dr. Chaomei Chen of Drexel University in the United States (11). It is primarily used to analyze the citation data in scientific literature and identify current research frontiers and development trends (12). In our study, CiteSpace (6.3.R1 Advanced) was utilized to analyze the institutions and countries/regions of the authors, references, and author keywords. The settings of CiteSpace were as follows: years per slice = 1 year, and the criteria for selecting institutions, references, and keywords include the g-index, a scale factor of *k* = 25, the strength of links measured by cosine similarity, and the scope of links measured within slices. The data for the authors’ countries/regions is based on the top 100% of the most cited or frequently occurring items from each slice to ensure all countries/regions are analyzed.

VOSviewer was developed by Van Eck and Waltman of Leiden University in the Netherlands. It is a Java-based software used for bibliometrics. It has strong graphic display capabilities and is suitable for large-scale data analysis (13). Therefore, we used VOSviewer (1.6.20.0) to analyze co-citation relationships and co-cited between authors and journals, and using full counting method to analyze the data of authors and journals.

### 2.3 Ethics statement

In this study, published papers were used rather than animal or human experiments. Therefore, institutional review board/ethics approval was not needed. There was no further contact with authors of the identified publications.

## 3 Results

### 3.1 Analysis of annual publications and citations

**Figure 2:**
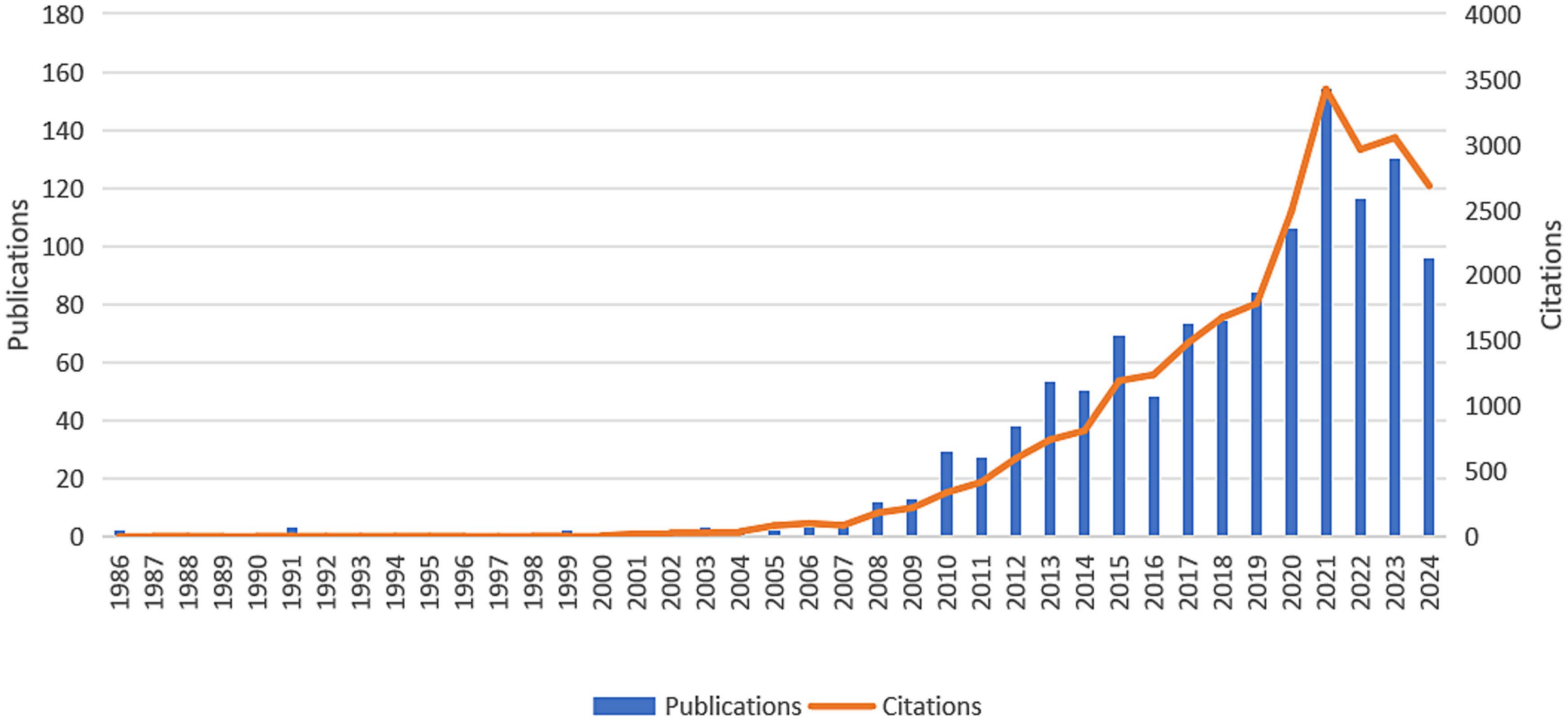
Annual number and trend of publications and citations.

In general, the number of publications in a given period determines the trend in a particular research field (14). As shown in Figure 2, the publications of TH for neonates were first appeared in 1986. During the first 20 years, the study on this field was in its infancy, with fewer than five annual publications. However, since 2007, the number of publications has increased year by year, and the number of citations per year has also risen rapidly. In other words, an increasing number of researchers focus on TH for neonates.

### 3.2 Analysis of countries/regions and institutions

**Figure 3:**
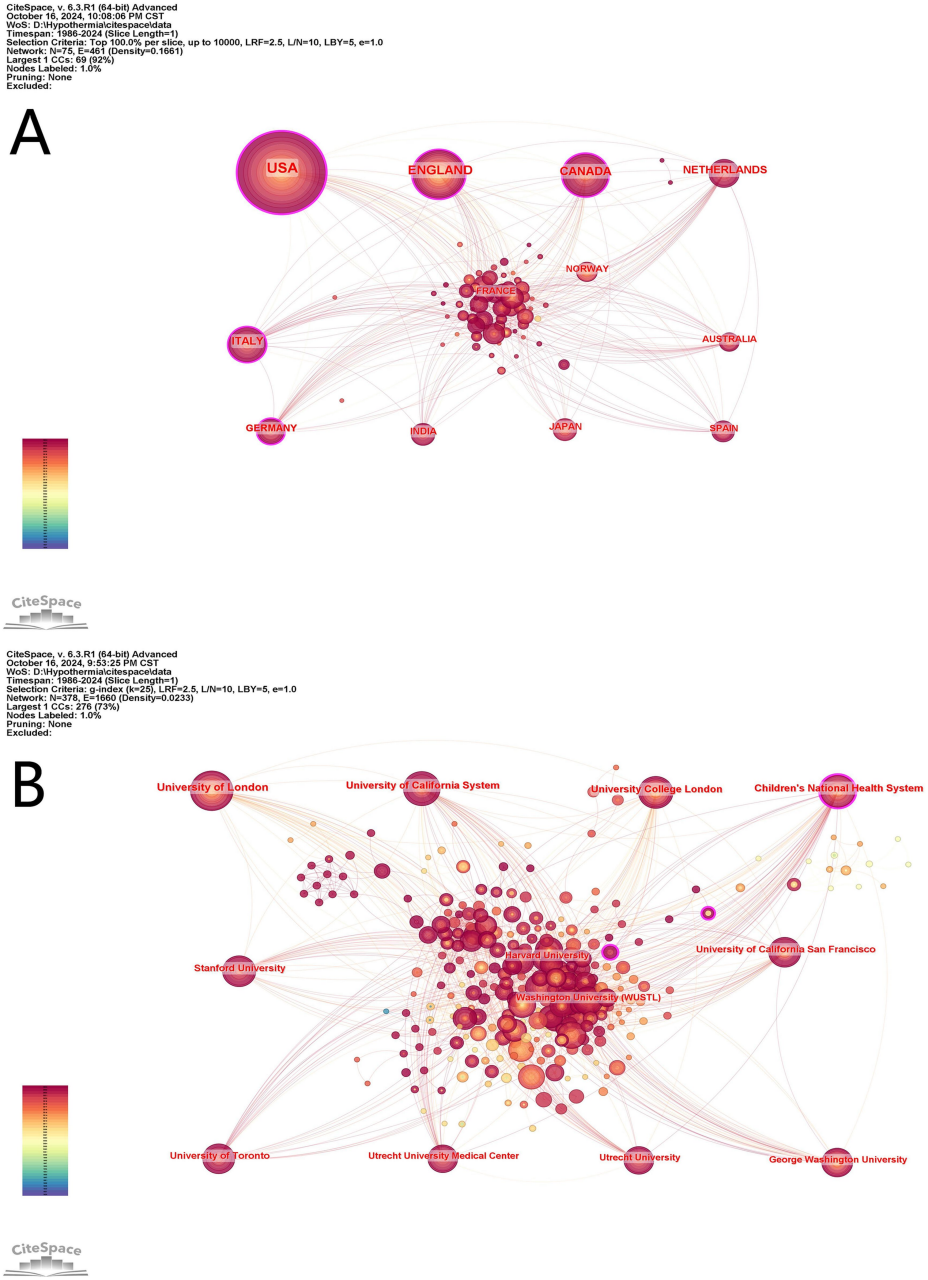
Cooperation map of countries/regions **(A)** and institutions **(B)** in therapeutic hypothermia for neonates. The size of the node is related with the co-occurrence frequencies, and the links is related with the co-occurrence relationships, and different node colors represent different years.

We analyzed the countries/regions and institutions using CiteSpace, and we found that the 1,199 articles were from 378 institutions of 75 countries/regions (Figure 3). The USA contributed the most publications (433, 25.15%), followed by England (172, 9.99%), Canada (132, 7.67%), Italy (88, 5.11%), and Netherlands (74, 4.30%) (Table 1). In top 10 countries/regions, the USA, India, and Germany were the earliest to participate the research, and most countries and regions exhibited high centrality (>0.1). As seen in the knowledge map of national cooperation networks, countries and regions cooperated relatively closely, especially in recent years (Figure 3A). Meanwhile, in 378 institutions, all the top 10 institutions published more than 40 articles (Table 1). Among them, the top publishing institution was University of London (79, 3.22%), followed by University of California System (63, 2.57%), University College London (60, 2.45%), Children’s National Health System (59, 2.41%), Stanford University (49, 2.00%). Five institutions of the top 10 are from the USA. As shown in Figure 3B, it is becoming increasingly common for institutions to collaborate over time, which greatly promotes progress in this area.

**Table 1:** The top 10 countries/regions and institutions involved in the study of therapeutic hypothermia for neonates.

Rank	Countries/regions	Count (%)	Year	Centrality	Institution	Count (%)	Year	Centrality
1	USA	433 (25.15%)	1986	0.26	University of London	79 (3.22%)	2007	0.1
2	ENGLAND	172 (9.99%)	2000	0.18	University of California System	63 (2.57%)	2008	0.08
3	CANADA	132 (7.67%)	2005	0.15	University College London	60 (2.45%)	2007	0.08
4	ITALY	88 (5.11%)	2009	0.13	Children’s National Health System	59 (2.41%)	2012	0.11
5	NETHERLANDS	74 (4.30%)	2008	0.1	Stanford University	49 (2.00%)	2012	0.03
6	GERMANY	53 (3.08%)	1999	0.12	University of California San Francisco	47 (1.92%)	2008	0.05
7	INDIA	49 (2.85%)	1995	0.03	University of Toronto	44 (1.79%)	2010	0.04
8	JAPAN	46 (2.67%)	2007	0	Utrecht University	42 (1.71%)	2009	0.05
9	AUSTRALIA	40 (2.32%)	2003	0.06	Utrecht University Medical Center	42 (1.71%)	2009	0.05
10	SPAIN	40 (2.32%)	2014	0.02	George Washington University	40 (1.63%)	2012	0.05

### 3.3 Analysis of authors and co-cited authors

**Figure 4:**
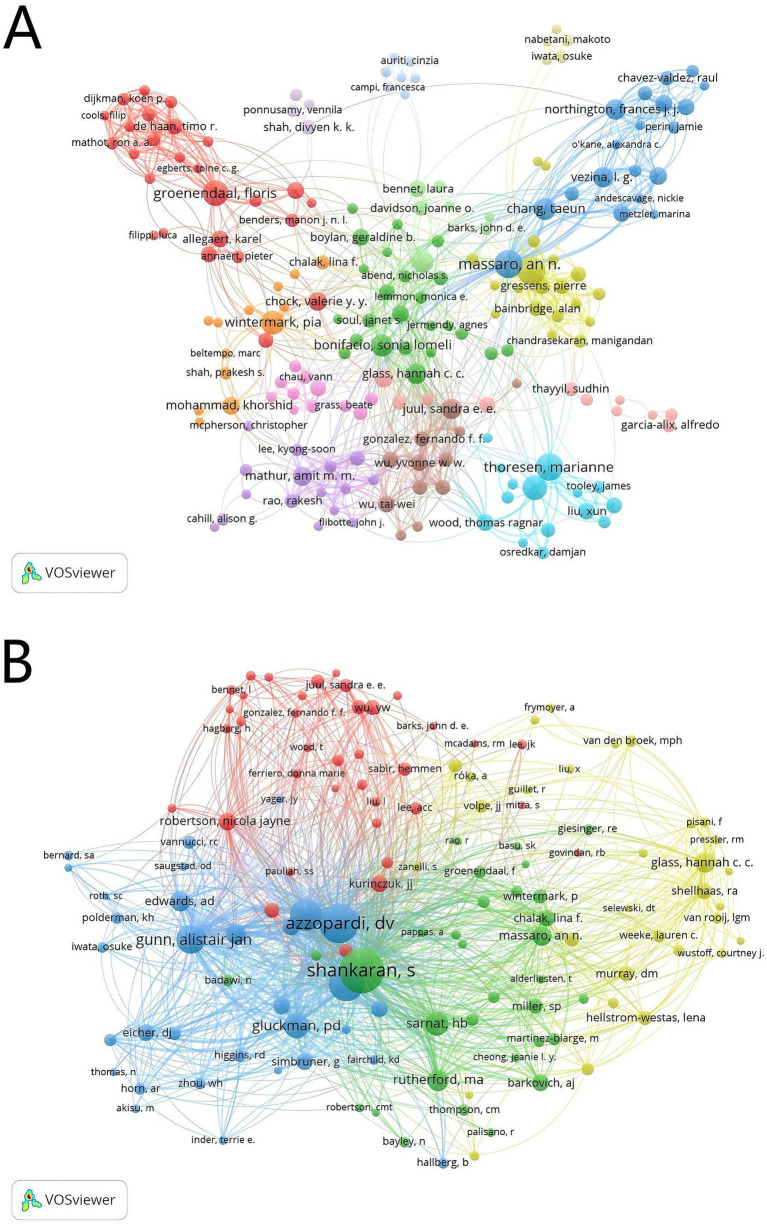
The co-occurrence authors **(A)** and the co-cited authors **(B)** in therapeutic hypothermia for neonates. The size of node is related with the author’s co-occurrence/co-cited frequencies, the link is related with the co-occurrence/co-cited relationship between authors, and different node colors represent different clusters.

A total of 4,843 authors were identified from 1,199 articles by VOSviewer; 211 of them published more than 5 articles. As shown in Table 2, Massaro, An N. N. from George Washington University, and Robertson. Nicola Jayne from University College London, had the most publications (*n* = 37); they are experts in pediatric neurology. Authors with more than five articles were shown in the visualization (Figure 4A), and most of them had cooperative relationships. In the result, 17,095 cited authors were found in the 1,199 articles, and 182 of them were cited more than 30 times. In Table 2, we can find that the most fequently cited author among co-cited author was Shankaran. S. (*n* = 1,149), followed by Azzopardi. DV (*n* = 894), Jacobs. Susan E. (*n* = 700), Thoresen. Marianne (*n* = 573) and Gunn. Alistair Jan (*n* = 475). The authors with citations at least 40 (*n* = 132) are shown in the knowledge map (Figure 4B). As shown in Figure 4B, all the authors had strong links with each other, particularly the top 10 cited authors, which promoted the research development in this field.

**Table 2:** The top 10 authors and co-cited authors in therapeutic hypothermia for neonates.

Rank	Author	Count	Co-cited author	Citations
1	Massaro, An N. N.	37	Shankaran, S	1,149
2	Robertson, Nicola Jayne	37	Azzopardi, DV	894
3	Thoresen, Marianne	35	Jacobs, Susan E.	700
4	Groenendaal, Floris	31	Thoresen, Marianne	573
5	Sabir, Hemmen	28	Gunn, Alistair Jan	475
6	Wintermark, Pia	26	Gluckman, Peter D.	379
7	Chang, Taeun	24	Sarnat, HB.	322
8	Gunn, Alistair Jan	24	Rutherford, Ma	262
9	Bonifacio, Sonia Lomeli	23	Edwards, A. David	240
10	Glass, Hannah C. C.	20	Glass, Hannah C. C.	238

### 3.4 Analysis of journals and co-cited journals

**Figure 5:**
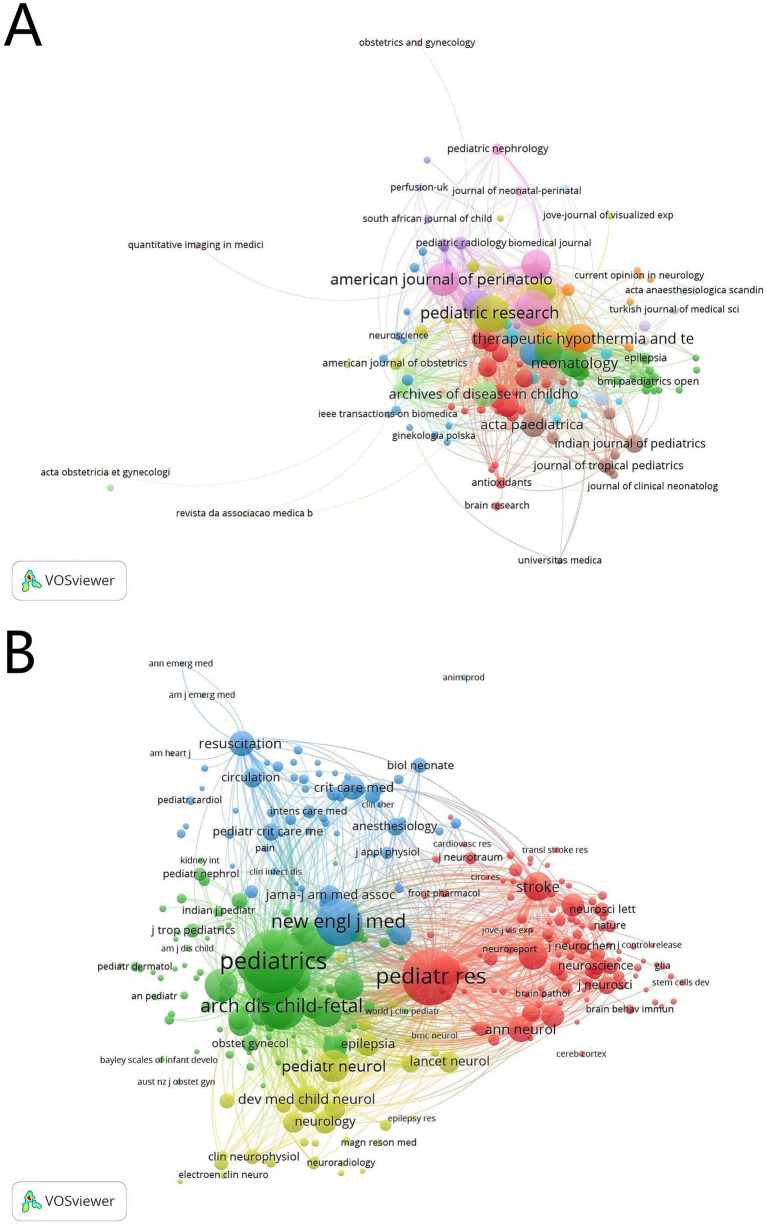
The co-occurrence journals **(A)** and the co-cited journals **(B)** in therapeutic hypothermia for neonates. The size of node is related with the journal’s co-occurrence/co-cited frequencies, the link is related with the co-occurrence/co-cited relationship between journals, and different node colors represent different clusters.

**Figure 6:**
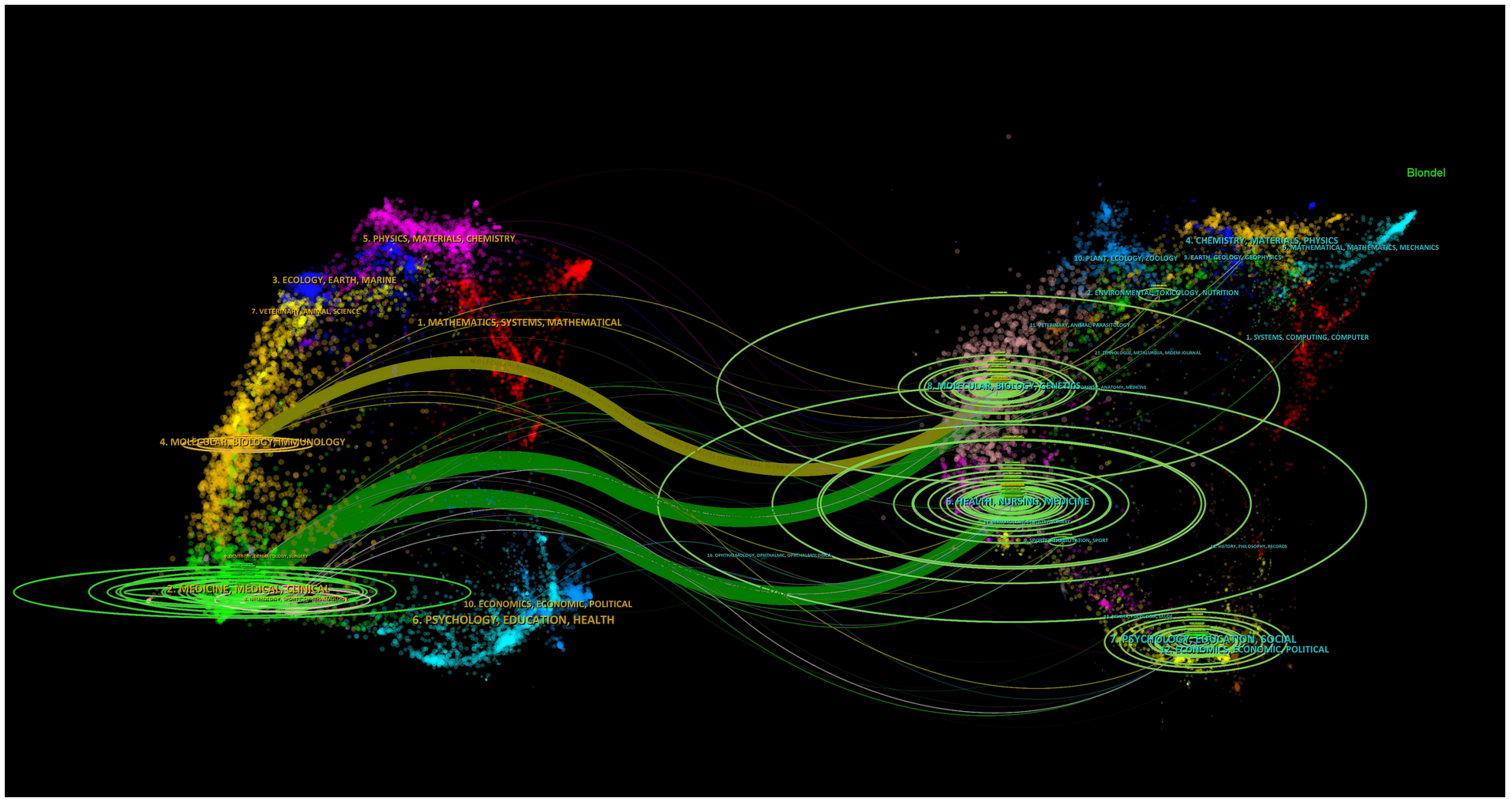
Dual-map overlay of journals related to therapeutic hypothermia for neonates. Citing journals appear on the left, cited journals appear on the right, and the colored path highlights citation relationships.

VOSviewer was used to analyze the number of publications and citation frequency of journals. We found that 1,199 articles were published in 337 journals (Figure 5A). In Table 3, we can find that *Pediatric Research* (*n* = 52), *Journal of Perinatology* (*n* = 50), and *American Journal of Perinatology* (*n* = 42) published more than 40 articles. Six of the top 10 journals belonged to JCR Q1. Figure 5A shows that the top 10 journals had active citation relationships, but there were no citation relationships in some journals. To analyze the co-cited journals, we found 4,458 co-cited journals, and 36 of them were co-cited at least 300 times. From Figure 5B, we found that *Pediatrics*, *Pediatric Research*, and *New England Journal of Perinatology, Journal of Pediatrics Medicine* were the important co-citation journals. As shown in Table 4, the top 10 co-cited journals belonged to JCR Q1: *New England Journal of Medicine* (*n* = 1,597) and *Lancet* (*n* = 823) had an impact factor (IF) above 90. *Pediatric Research*, *Journal of Perinatology, Journal of Pediatrics*, and *Pediatric Neurology* were included in both the top 10 journals and the top 10 co-cited journals, indicating that they were the important journals in the field of TH for neonates. In addition, we used dual-map overlay to analyze the relationship between citing journals and cited journals using CiteSpace (15). From Figure 6, two green and one yellow citation paths can be identified. The two green paths show that the articles published in the areas of molecular/biology/genetics and health/nursing/medicine were always cited by medicine/medical/clinical, and the yellow one indicates the researches in molecular/biology/genetics were frequently cited by molecular/biology/immunology.

**Table 3:** The top 10 journals in therapeutic hypothermia for neonates.

Rank	Journal	Count	IF	JCR
1	Pediatric Research	52	3.1	Q1
2	Journal of Perinatology	50	2.4	Q1
3	American Journal of Perinatology	42	1.5	Q2
4	Journal of Pediatrics	33	3.9	Q1
5	Frontiers in Pediatrics	32	2.1	Q2
6	Therapeutic Hypothermia and Temperature Management	32	0.8	Q4
7	Seminars in Fetal and Neonatal Medicine	29	2.9	Q1
8	Neonatology	29	2.6	Q1
9	Journal of Maternal-Fetal and Neonatal Medicine	26	1.7	Q3
10	Pediatric Neurology	25	3.2	Q1

**Table 4:** The top 10 co-cited journals in therapeutic hypothermia for neonates.

Rank	Journal	Citations	IF	JCR
1	Pediatrics	2,925	3.9	Q1
2	Pediatric Research	2,319	3.1	Q1
3	Journal of Pediatrics	1988	3.9	Q1
4	New England Journal of Medicine	1,597	96.2	Q1
5	Archives of Disease in Childhood-fetal and Neonatal Edition	1,576	3.9	Q1
6	Journal of Perinatology	973	2.4	Q1
7	Lancet	823	98.4	Q1
8	Acta Paediatrica	816	2.4	Q1
9	Cochrane Database of Systematic Reviews	816	8.8	Q1
10	Pediatric Neurology	746	3.2	Q1

### 3.5 Analysis of references

**Figure 7:**
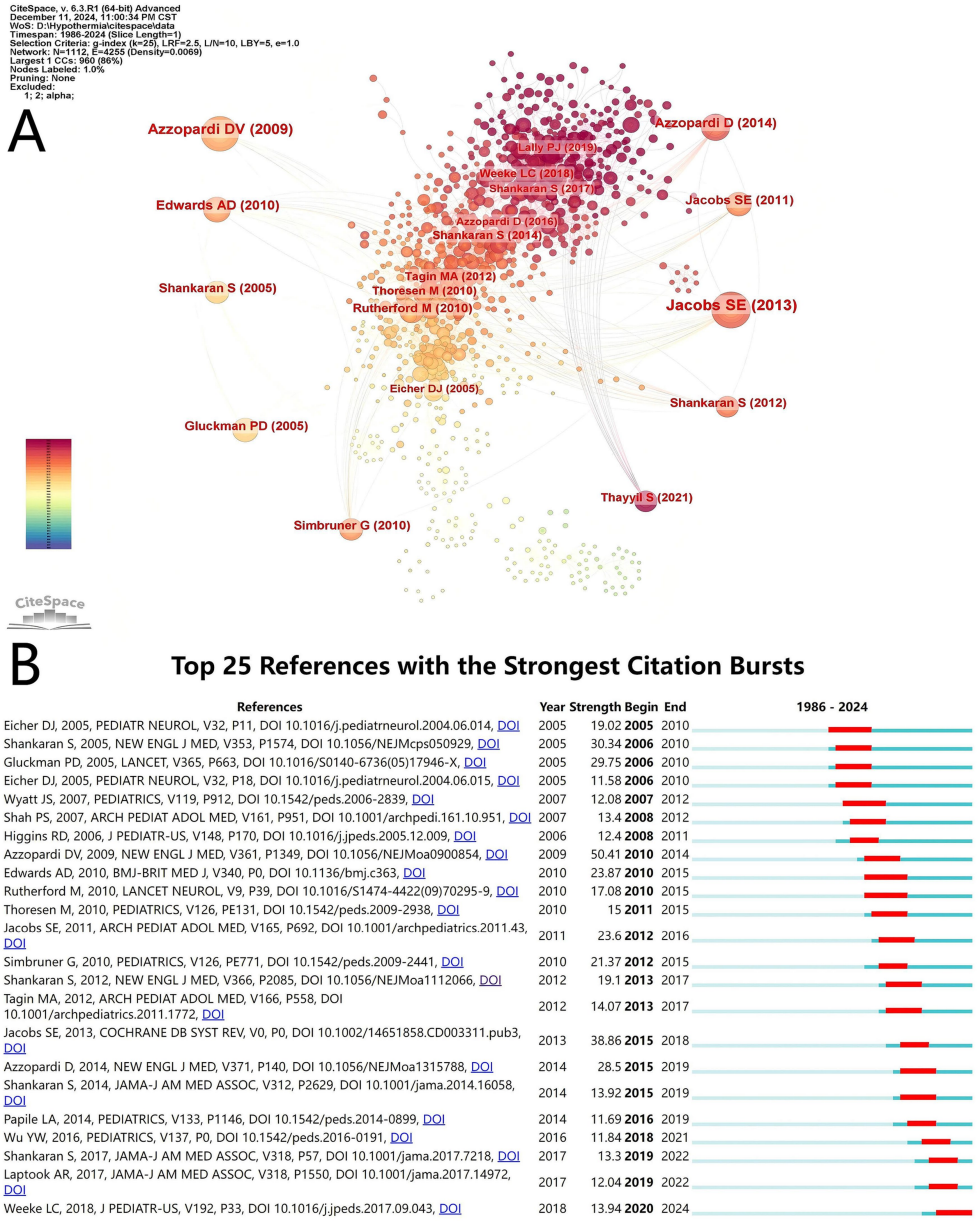
Reference visualization. **(A)** Co-cited network map of references related to therapeutic hypothermia for neonates research. The size of the node is related with the co-cited frequencies, and the links is related with the co-cited relationships, and different node colors represent different years. **(B)** The top 25 references with the strongest citation bursts. The light blur bar indicates the reference has not been published; the dark blue bar indicates the reference has been published; the red bar indicates citation bursts.

We used CiteSpace to analyze the references in our study. As a result, 1,112 co-cited references were found regarding the research on TH for neonates, showing close co-citation relationships between them (Figure 7A). In Table 5, we can see that all of the top 10 references were cited at least 46 times, with two of them were more than 109 times. The review article “Cooling for newborns with hypoxic ischemic encephalopathy” (16), published in Cochrane Database of Systematic Reviews by Jacobs SE in 2013, determined the effect of TH for neonates with encephalopathic asphyxia. The authors found that the benefits of cooling on survival and neurodevelopment outweigh the short-term risk. “Effects of hypothermia for perinatal asphyxia on childhood outcomes” (17) and “Childhood outcomes after hypothermia for neonatal encephalopathy” (18) were randomized controlled trials that focused on childhood outcomes related to hypothermia. A total of 194 studies with burst co-cited at least 2 years were identified through burst analysis using CiteSpace, and 25 co-cited studies with the strongest citations were selected (Figure 7B). It showed that the first burst co-cited references was began in 2005; one of the top 25 references with the strongest citation bursts is still citation bursts. The strength of 25 references was greater than 11.58, with the highest being 50.41. They are the important pieces of literature in TH for neonates, indicating the research hotspots in various stages.

**Table 5:** The top 10 references in therapeutic hypothermia for neonates.

Rank	First author	Title	Journal	Year	citations
1	Jacobs SE	Cooling for newborns with hypoxic ischaemic encephalopathy	Cochrane Database of Systematic Reviews	2013	133
2	Azzopardi DV	Moderate hypothermia to treat perinatal asphyxial encephalopathy	New England Journal of Medicine	2009	109
3	Azzopardi DV	Effects of hypothermia for perinatal asphyxia on childhood outcomes	New England Journal of Medicine	2014	69
4	Edwards AD	Neurological outcomes at 18 months of age after moderate hypothermia for perinatal hypoxic ischaemic encephalopathy: synthesis and meta-analysis of trial data	BMJ-British Medical Journal	2010	64
5	Jacobs SE	Whole-body hypothermia for term and near-term newborns with hypoxic–ischemic encephalopathy: a randomized controlled trial	Archives of Pediatrics and Adolescent Medicine	2011	55
6	Shankaran S	Whole-body hypothermia for neonates with hypoxic–ischemic encephalopathy	New England Journal of Medicine	2005	52
7	Gluckman PD	Selective head cooling with mild systemic hypothermia after neonatal encephalopathy: multicentre randomized trial	Lancet	2005	51
8	Rutherford M	Assessment of brain tissue injury after moderate hypothermia in neonates with hypoxic-ischaemic encephalopathy: a nested substudy of a randomized controlled trial	Lancet Neurol	2010	46
9	Shankaran S	Childhood outcomes after hypothermia for neonatal encephalopathy	New England Journal of Medicine	2012	46
10	Simbruner G	Systemic hypothermia after neonatal encephalopathy: outcomes of neo.nEURO.network RCT	Pediatrics	2010	46

### 3.6 Analysis of keywords

**Figure 8:**
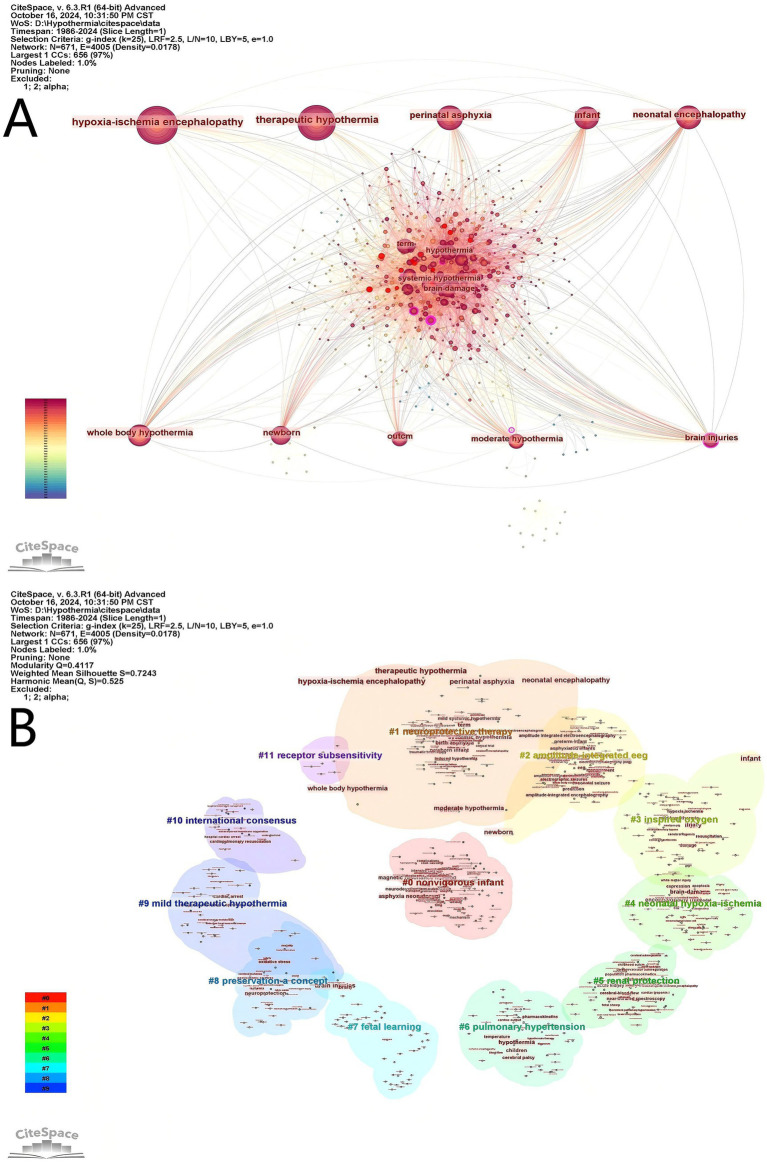
Keyword visual analysis. **(A)** Cooperation map of keywords in therapeutic hypothermia for neonates. **(B)** Cluster map of keywords in related to therapeutic hypothermia for neonates research.

**Figure 9:**
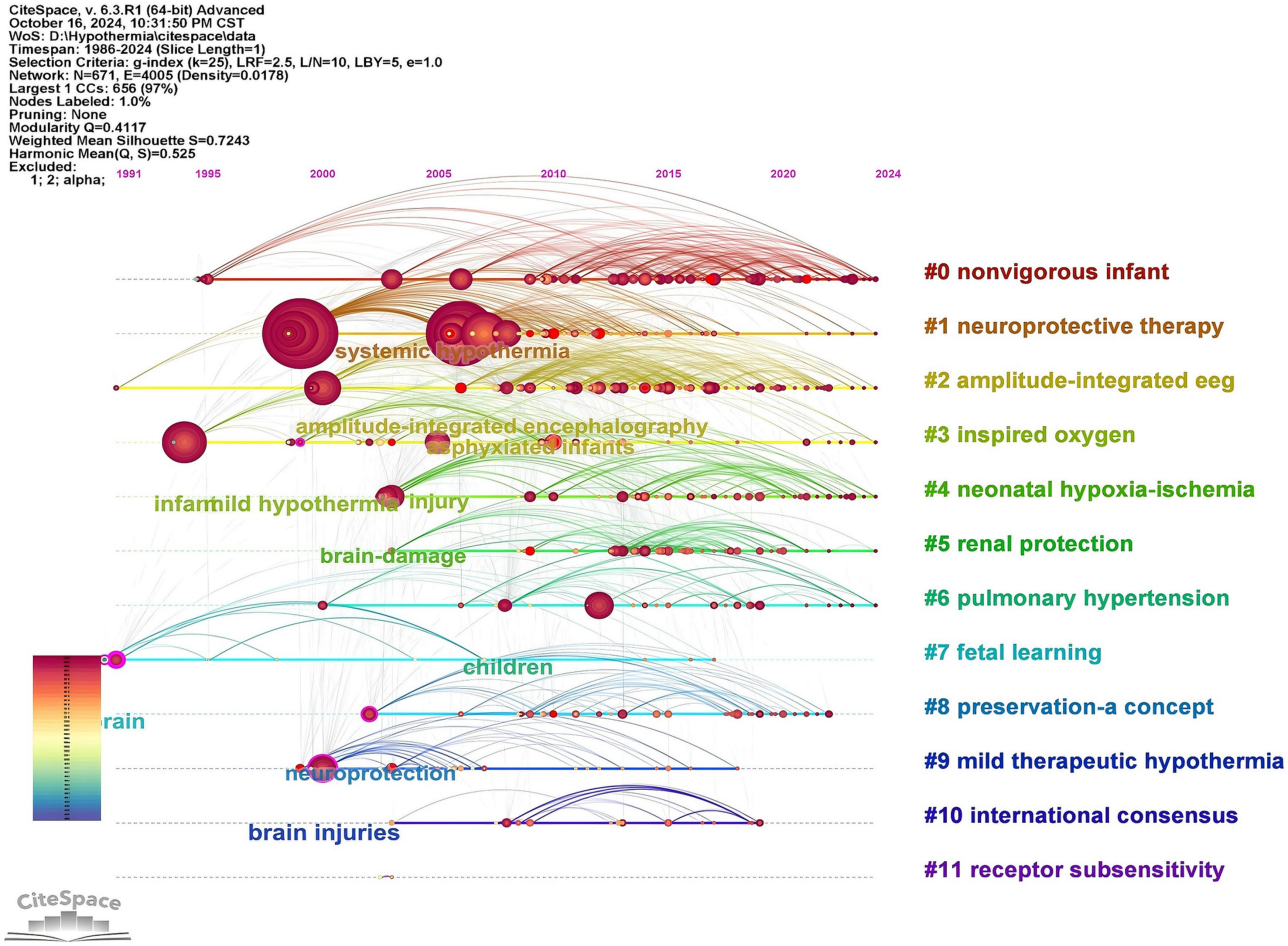
Timeline map of keywords in therapeutic hypothermia for neonates. The horizontal axis represents clustering, the vertical axis represents time, and the node size is related with the co-occurrence frequencies.

**Figure 10:**
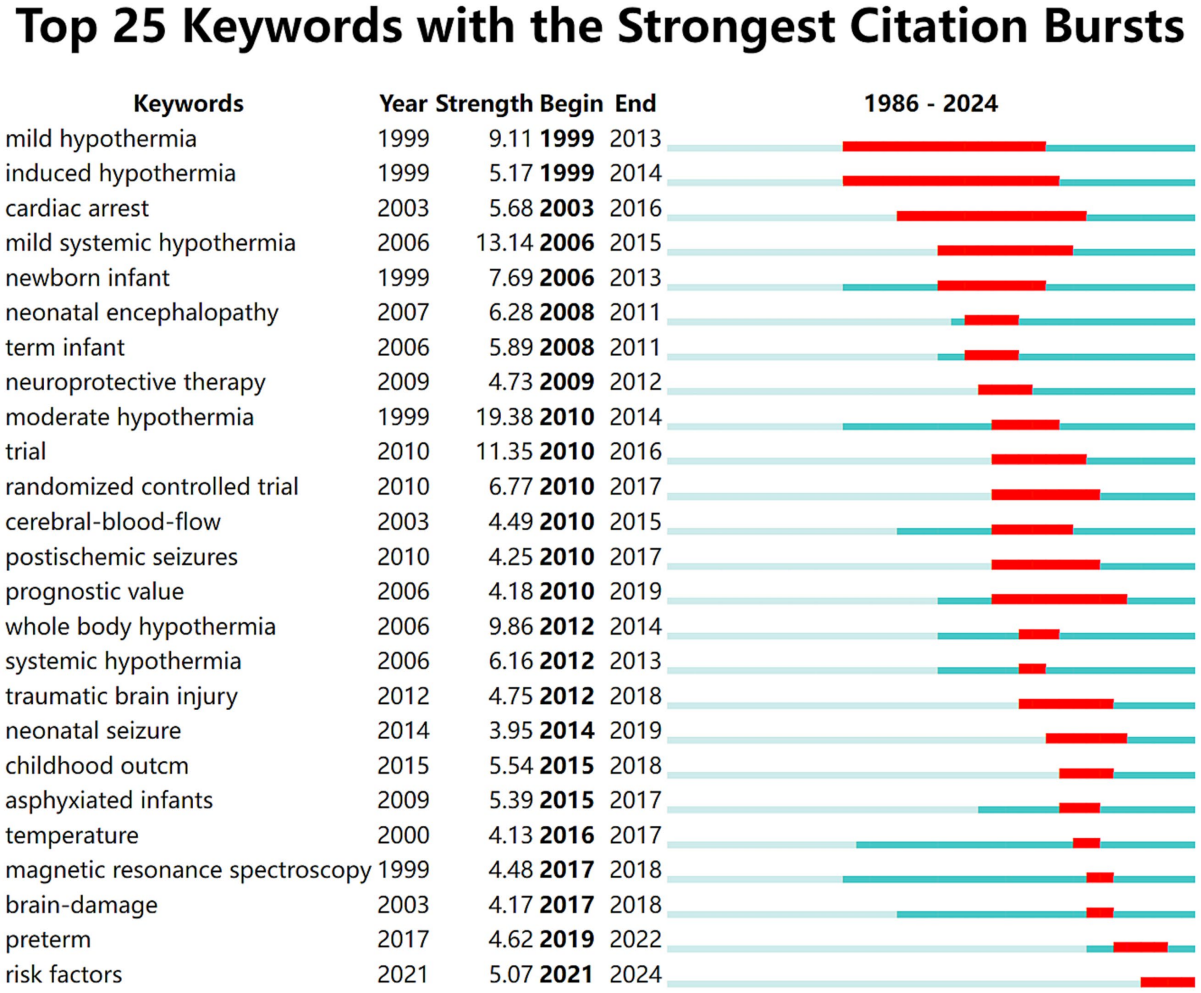
The top 25 keywords with the strongest citation bursts. The light blur bar indicates the keyword has not appeared in the articles; the dark blue bar indicates the keyword has appeared in the articles; the red bar indicates citation bursts.

CiteSpace was used to analyze the author keywords. At the current threshold setting, a network map with 671 nodes, 4,005 connections and a density of 0.0178 was obtained (Figure 8A). It showed that 671 keywords were identified in 1,199 articles; “hypoxia-ischemia encephalopathy” (*n* = 580), “therapeutic hypothermia” (*n* = 507), and “perinatal asphyxia” (*n* = 296) had high frequencies, but 252 of them appeared only once. As shown in Figure 8B, all keywords were divided into 12 clusters, using title words to label clusters: #0 “non-vigorous infant,” #1 “neuroprotective therapy,” #2 “amplitude-integrated eeg,” #3 “inspired oxygen,” #4 “neonatal hypoxia-ischemia,” #5 “renal protection,” #6 “pulmonary hypertension,” #7 “fetal learning,” #8 “preservation-a concept,” #9 “mild therapeutic hypothermia,” #10 “international consensus,” #11 “receptor subsensitivity.” The clustering structure was validated as appropriate and credible by a Modularity Q of 0.4117 and a Silhouette S of 0.7243, ensuring that the keyword cluster labels were consistent with the top 10 keywords. Timeline map of keywords shows that clusters #0, #1, #2, #3, #5, and #6 were the important areas in the research, while “renal protection,” and “pulmonary hypertension” were the emerging research areas in TH for neonates (Figure 9). As displayed in Figure 10 the top 25 keywords with strongest citation bursts. “Mild hypothermia,” “induced hypothermia,” and “cardiac arrest” had longest burst time, each exceeding over 10 years. They were early research hotspots in this field. “Moderate hypothermia” had the highest strength, and was the hotspot from 2010 to 2014. In the field of TH for neonates, researchers initially focused on the research of “cardiac arrest,” “neonatal encephalopathy,” “term infant,” and “neuroprotective therapy.” Over time, researchers paid more attention to “childhood outcome,” “neonatal seizure,” “preterm,” and “risk factors” in recent years.

## 4 Discussion

In this study, a bibliometric analysis of TH for neonates was conducted using CiteSpace (6.3.R1 Advanced) and VOSviewer (1.6.20.0). Research on TH for neonates began in 1986, and has developed rapidly in the last 17 years. The number of publications and citations were both increased year by year. The USA, England, and Canada are the important counties which published over 100 articles in this field, while the USA, India, and Germany were the earliest counties to participate in the study. A total of 378 institutions made a contribution in this field. Among them, five of the top 10 institutions were from the USA, and University of London had the most publications. Animal experiments from the University of London have shown that TH combined with melatonin has a good neuroprotective effect on piglets with asphyxia (19), but TH has no effect on inflammation-sensitized hypoxic–ischemic piglets (20). By analyzing authors and cited authors, Massaro. An N. N., Robertson. Nicola Jayne, and Thoresen, Marianne are the three authors with the most publications, while Shankaran. S, Azzopardi. DV, and Jacobs. Susan E. are the three most cited authors. They are important pediatric specialists, especially in the field of TH for neonates. In terms of journal analysis, *Pediatric Research* and *Journal of Perinatology* were the major journal which published more than 50 studies on TH for neonates, while *Pediatrics* and *Pediatric Research* were cited over 2000 times. *Pediatric Research* is prominent for original translational research articles on the causes and treatments of childhood diseases and developmental disabilities. *Journal of Perinatology* mainly publishes articles focused on improving maternal, fetal, and, neonatal care. *Pediatrics* emphasizes original researches, clinical observations, and feature articles in the broadly defined field of pediatrics. These three journals are important in medical field. Meanwhile, we found that the researches in this field focused on fundamental and clinical studies through dual-map overlay.

The analysis of references and keywords can show the hotspots and frontiers of research. TH is recommended as a routine treatment for moderate to severe hypoxic–ischemic encephalopathy (HIE) (21), as it can improve neurological prognosis in survivors (22). Studies have shown that both whole-body hypothermia and selective head cooling with mild systemic hypothermia can reduce the mortality of infants with perinatal asphyxia (23, 24). A meta-analysis indicated that TH in neonates was associated with a reduction in death and neurological impairment at 18 months (25), and Azzopardi. DV found that it could reduce the risk of cerebral palsy and severe disability in childhood (17). Packer CH pointed out through a cost–benefit analysis that TH can improve the outcome of neonates with severe perinatal asphyxia (26). However, some children with mild hypoxic–ischemic encephalopathy in the neonatal period experience neurodevelopmental impairment, and a study from 35 countries found that most neonatal practitioners would consider TH for infants with mild HIE (27). A study showed that TH can reduce the brain injury in neonates with mild HIE (28). At present, TH is recommended for the neonates with a gestational age over 36 weeks and within 6 h of birth in most countries, but babies born in the non-cooling center are prone to delay TH (29). In order to benefit more neonates, researchers studied cooling outside the therapeutic window and TH for preterm infants. A multicenter randomized controlled trial showed that TH initiated 6 to 24 h after birth can reduce the risk of death or disability in term infants with HIE compared to no cooling (30). In terms of preterm infants, one retrospective study concluded that TH is safe for preterm infants with a gestational age of 34–35 weeks (31); however, other studies suggested that TH for preterm infants younger than 36 weeks was more likely to be associated with abnormal coagulation, severe intracranial hemorrhage, and higher mortality (32, 33).

In recent years, research in this field has paid more attention to how to improve the effect of TH, and reduce the incidence of adverse events. A study on temperature monitoring during TH found that overcooling and hypotension were more likely to occurred in newborns with esophageal temperature monitoring (34). And a clinical trial showed that reaching a target temperature within 6 h of birth, whether early (≤4 h) or late (>4 h), was not associated with neurodevelopmental outcomes (35). For neonates with moderate or severe HIE, 72 h of hypothermia treatment is better than 48 h, which can improve oxidation conditions, reduce neuron-specific enolase value, and improve neurobehavior and development (36). An animal experiment showed that neither longer nor deeper cooling could improve the prognosis of neonatal HIE rats (37). However, clinical trials showed that, compared to hypothermia at 33.5°C for 72 h, longer or deeper cooling did not reduce mortality and increased complications of TH in term neonates with moderate to severe HIE (38, 39). In terms of rewarming, a prospective randomized controlled trial showed that the short-term clinical effect of rewarming for 10 h is better than that of rewarming for 25 h, and that extending the rewarming time is not conducive to the formation of a normal sleep–wake cycle (40). Regarding feeding during TH, a randomized controlled trial suggested that early enteral nutrition may be feasible and safe to implement, and it can shorten the length of hospital stay (41). In terms of adverse reactions of TH, a study from Brazil showed that neonates undergoing TH frequently experienced seizures, especially electroconvulsive seizures (42). Infants who had seizures during TH were at high risk for having seizures again during rewarming (43). Because infants with seizures had poor neurological outcome (44), it is necessary to monitor amplitude-integrated EEG during TH and after rewarming. Phenobarbital is still the primary treatment for neonatal seizures, and it is safe to discontinue phenobarbital after the seizure was controlled (45). There was no significant effect on neurological development or seizure incidence at 24 months of age (46). Meanwhile, acute kidney injury is common in neonates with HIE, and TH cannot alter its incidence; therefore, long-term follow-up of renal function is required (47). A prospective observational study found that acute kidney injury was a better predictor of unfavorable long-term outcome in neonates with HIE treated with TH (48). However, although TH has become a routine treatment for moderate to severe neonatal HIE, nearly half of the infants treated with TH still die or have severe disability (49). Therefore, it is important to predict the unfavorable prognosis early, as this can facilitate timely rehabilitation treatment. A clinical study showed that the evolution of amplitude-integrated EEG background pattern was a better predictor of the prognosis of neonates treated with TH than amplitude-integrated EEG background pattern (50). A study from two neonatal intensive care units suggested that elevated plasma Tau levels were associated with the degree of brain damage in neonates with HIE treated with TH and could serve as a marker of neonatal brain damage (51). Pineles et al. pointed out that central nervous system-derived exosomes can be used as a biological indicator of brain injury severity and response to TH (52). The study from Catherine et al. (53) showed that S100 calcium-binding protein B and Neuron specific enolase have no significant relationship with the neurodevelopmental outcome of neonates treated with TH. Magnetic resonance imaging diffusion-weighted imaging, and magnetic resonance spectroscopy are important imaging methods for predicting nervous system injury in neonates with perinatal asphyxia treated with TH (54, 55). And a report from the Children’s Hospital Neonatal Consortium found that magnetic resonance abnormalities in neonates with mild HIE treated with TH were associated with complications during and after hypothermia (56).

Despite treating with TH, some neonates with perinatal asphyxia experience severe neurodevelopmental disorders. In order to improve the neuroprotective effect of TH, more and more researchers have paid attention to the combination of TH with other therapies. A meta-analysis showed that TH combined with melatonin has a neuroprotective effect (57), and could improve long-term neurological outcomes (58). A meta-analysis suggested that erythropoietin improves outcomes in neonates with HIE who cannot be treated with TH (59). However, a multicenter study showed that TH combined with erythropoietin increased the risk of thrombosis (60) and did not reduce the incidence of local brain injury, but subacute brain injury was more common (61). In a randomized controlled trial, Kumar C.’s team found that TH combined with magnesium sulfate did not improve neonatal mortality or the outcome of severe neurodevelopmental disorders (62). An animal experiment found that TH combined with allopurinol had neuroprotective effect on rat pups with HIE (63) and a multicenter randomized controlled trial demonstrated the positive effects of allopurinol combined with TH on improving neurocognition in neonates with moderate-to-severe HIE (49). In addition, researchers found that exendin-4, cannabidiol, and inhaled argon at concentrations of 40–50% could enhance the neuroprotective effect of TH in animal experiments (64–66).

In summary, the hotspots and frontiers of TH for neonates include how to manage the infant with perinatal asphyxia treated with TH; whether to expand TH beneficiary groups, such as infants with mild HIE and preterm infants; to seek effective combination therapy to enhance neuroprotective effect.

However, this study has some limitations. Firstly, the articles were obtained only from WOSCC, rather than other databases such as PubMed, so some related articles were inevitably missed. Secondly, we included only English-language studies, while the studies in other languages were excluded. Thirdly, some recently published high quality articles may not have received sufficient attention because of low citation counts. Lastly, the visualization tools we used may not be able to fully capture the nuances of specific research trends.

## 5 Conclusion

In conclusion, the present study is a bibliometric analysis and visualization research on therapeutic hypothermia for neonates, and providing hotspots and frontiers for this field. The number of publications and citations in this field increased year by year, especially in the last two decades. The USA was the most productive country, while University of London had the most publications. Massaro, An N. N., and Shankaran. S were the most contributing and most influential authors, respectively. The most productive journal was Pediatric Research, and the most co-cited journal was Pediatrics. The hotspots were TH for neonates with perinatal asphyxia and strategies for managing these infants. Recently, researchers focused on how to expand the benefits for the neonatal population and enhance neuroprotective effects, in order to improve the neurological outcomes of newborns. It may be the future research trend of neonatologists.

## Data Availability

The original contributions presented in the study are included in the article/supplementary material, further inquiries can be directed to the corresponding authors.

## Footnotes

^1^ https://www.ncbi.nlm.nih.gov/mesh
